# First case report of neoadjuvant gemcitabine and S-1 for locally advanced unresectable duodenal adenocarcinoma

**DOI:** 10.1186/s40792-022-01453-2

**Published:** 2022-05-19

**Authors:** Jiro Kimura, Kenta Sui, Motoyasu Tabuchi, Takahiro Murokawa, Shinya Sakamoto, Jun Iwata, Manabu Matsumoto, Takehiro Okabayashi

**Affiliations:** 1 Department of Gastroenterological Surgery at Kochi Health Sciences Center, 2125-1 Ike, Kochi-City, Kochi 781-8555 Japan; 2 Department of Diagnostic Pathology at Kochi Health Sciences Center, 2125-1 Ike, Kochi-City, Kochi 781-8555 Japan

**Keywords:** Duodenal adenocarcinoma, Neoadjuvant chemotherapy, Gemcitabine, S-1

## Abstract

**Background:**

The usefulness of neoadjuvant chemotherapy for patients with duodenal adenocarcinoma remains unclear. We report the case of a successfully resected duodenal adenocarcinoma managed by neoadjuvant chemotherapy using gemcitabine and S-1.

**Case presentation:**

A 72-year-old female presented with a one-week history of abdominal bloating and vomiting after meals. Esophagogastroduodenoscopy revealed a circumferential epithelial lesion in the second portion of the duodenum. Abdominal computed tomography scan revealed thickened walls and narrowing of the duodenum. Further, an adenocarcinoma was noted on biopsy. Though she was diagnosed with duodenal adenocarcinoma, pancreatic cancer could not be completely ruled out. Therefore, she underwent neoadjuvant chemotherapy using gemcitabine and S-1 after bypass surgery. After six chemotherapy cycles, the tumor significantly reduced in size. Further, lymph nodes and distant metastases were not noted on abdominal computed tomography. The patient underwent pancreaticoduodenectomy. Pathological examination revealed a 0.5-mm lesion and surrounding fibrosis at the duodenum, distal from the ampulla of Vater and the pancreas. Her postoperative course was almost uneventful, and she was discharged on the 31st postoperative day. The patient was followed up and had no tumor recurrence at 24 months after surgery.

**Conclusion:**

Neoadjuvant chemotherapy with gemcitabine and S-1 was useful in reducing the size of a duodenal adenocarcinoma. This finding would aid physicians in managing patients that present with a similar presentation.

## Background

Duodenal carcinoma (DC) is an uncommon malignancy, accounting for only 0.4% of gastrointestinal cancers [1]. Because patients with DCs present with nonspecific symptoms, its diagnosis is challenging, and patients often present with advanced disease. Its management includes complete surgical resection when feasible. Though adjuvant therapy is recommended in patients with DC [2], the role of neoadjuvant therapy remains unclear, especially in patients with locally advanced unresectable DC.

The usefulness of neoadjuvant chemotherapy has been reported in various types of cancers such as pancreatic and rectal cancers [3, 4]. We report a case of a successfully resected duodenal adenocarcinoma managed by neoadjuvant chemotherapy using gemcitabine and S-1.

## Case presentation

**Fig. 1:**
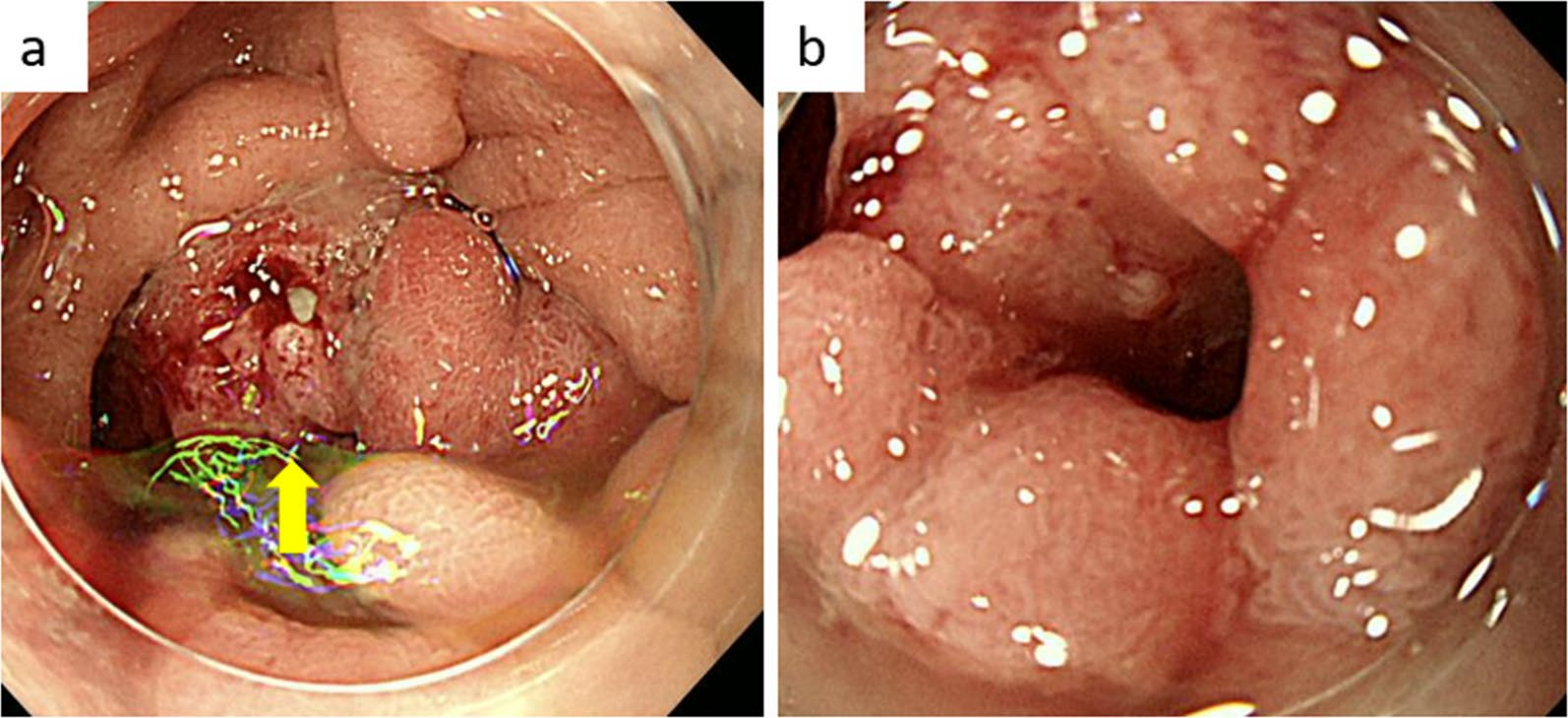
Esophagogastroduodenoscopy findings. **a** Circumferential epithelial lesion was found at the second portion of the duodenum (arrow). **b** The lumen was prominently narrowing and almost obstructed

**Fig. 2:**
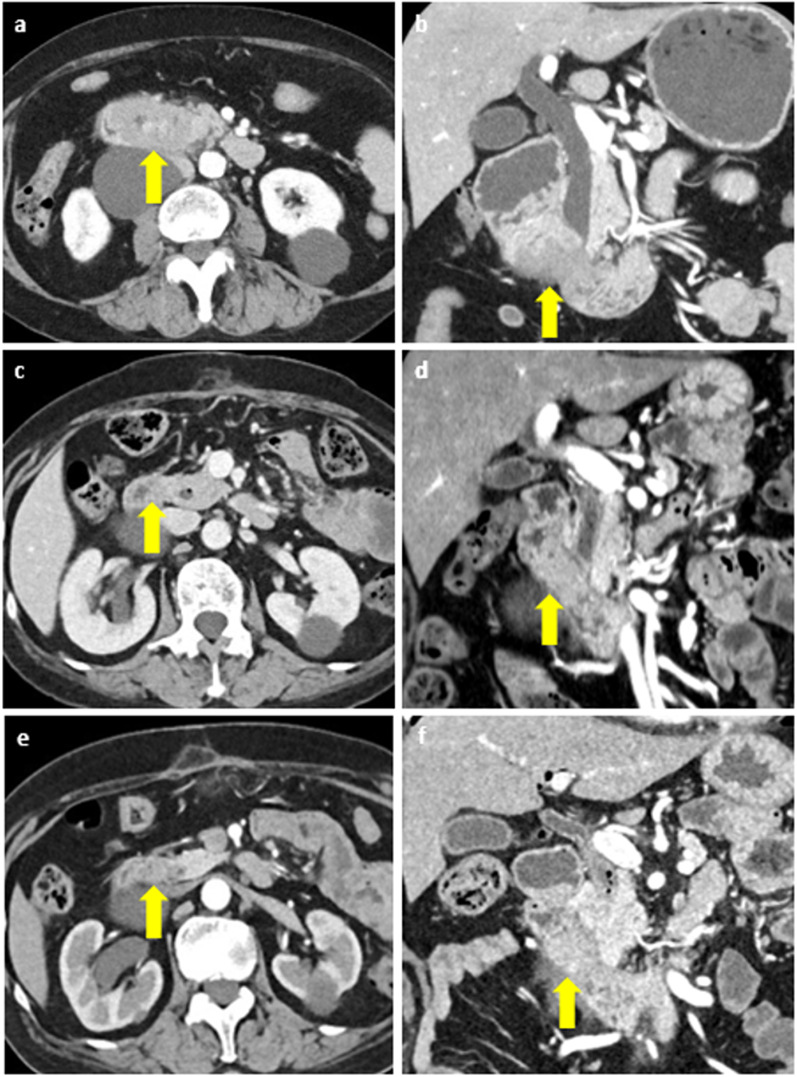
Contrast enhanced abdominal computed tomography. **a** Before chemotherapy, axial view. The tumor was at the second portion of the duodenum (arrow). The lumen of the duodenum and the common bile duct was almost obstructed. **b** Before chemotherapy, coronal view. **c** After 3 courses of chemotherapy, axial view. The size of the tumor had decreased (arrow). **d** After 3 courses of chemotherapy, coronal view. **e** After 6 courses of chemotherapy, axial view. The tumor had extremely shrunken and was not detected (arrow). **f** After 6 courses of chemotherapy, coronal view

**Fig. 3:**
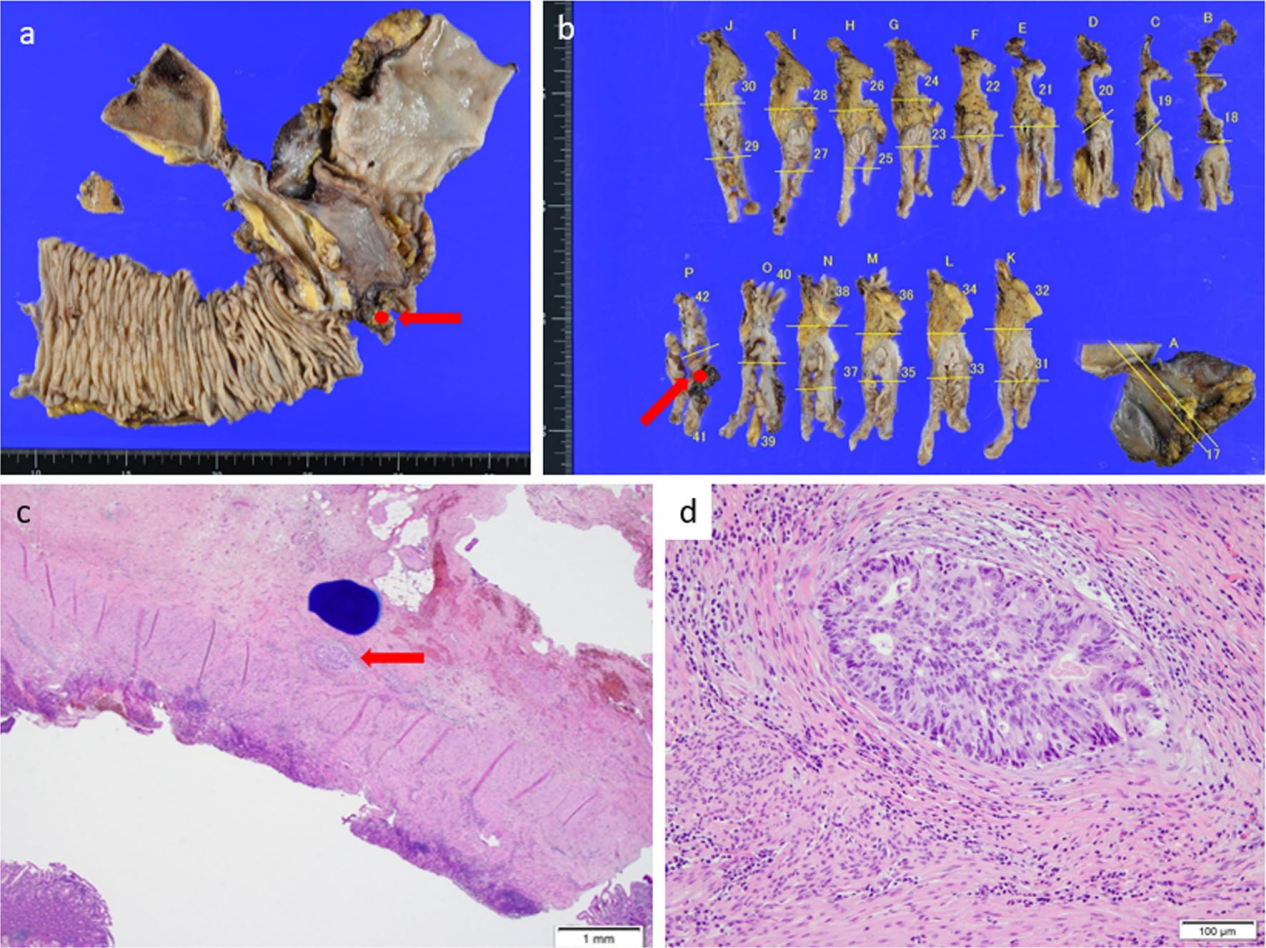
Specimen. **a** Macroscopic findings. The viable tumor was located at the duodenum away from the ampulla of Vater (arrow). **b** Cut surface of the specimen. (The tumor was pointed by arrow). **c** Microscopic findings. H.E. staining, 40 ×. A cluster of tumor cells was pointed by arrow. **d** H.E. staining, 400 ×

A 72-year-old female presented with a one-week history of abdominal bloating and vomiting after meals. She was previously diagnosed with diabetes mellitus. Esophagogastroduodenoscopy revealed a circumferential epithelial lesion in the second portion of the duodenum (Fig. 1). Abdominal computed tomography (CT) scan revealed thickened walls and narrowing of the duodenum (Fig. 2a, b). Further, an adenocarcinoma was noted on biopsy. Though she was diagnosed with a duodenal adenocarcinoma, pancreatic cancer could not be completely ruled out. Therefore, she underwent neoadjuvant chemotherapy of gemcitabine and S-1 (GS) after bypass surgery (choledochojejunostomy and gastrojejunostomy). For each course, she received gemcitabine infusion (1000 mg/m^2^) on the first and eighth days. S-1 was administered orally twice daily (80 mg/day) for 2 weeks. The size of the tumor gradually decreased without any lymph nodes or distant metastases (Fig. 2c, d). After six courses of chemotherapy, an abdominal CT scan revealed that the tumor had significantly reduced in size (Fig. 2e, f). No adverse events were observed during her chemotherapy regimen. The patient then underwent pancreaticoduodenectomy. Pathological examination revealed a 0.5-mm lesion with surrounding fibrosis at the duodenum, distal from the ampulla of Vater and the pancreas (Fig. 3).

Except for the development of a postoperative grade B pancreatic fistula, her postoperative course was uneventful. She was discharged from the hospital on postoperative day 31 [5]. At a follow-up examination in the outpatient clinic at postoperative 24 months, no evidence of recurrence was detected.

## Discussion

To the best of our knowledge, this is the first study reporting the use of neoadjuvant GS in patients with duodenal adenocarcinoma. Interestingly, it was markedly effective in a 72-year-old Japanese woman.

It was sometimes challenging for surgeons to differentiate duodenal adenocarcinoma from ampullary and pancreatic head cancers. Generally, it is pathologically impossible to differentiate these three cancers using biopsy alone. The only key feature differentiating the three would be the tumor location. Creating a preoperative diagnosis is essential because treatment plans for these tumors are different. In fact, we could not rule out pancreatic cancer preoperatively. Therefore, neoadjuvant GS treatment after bypass surgery was used in the case to manage pancreatic cancer, which had a worse prognosis [6].

Studies have shown that neoadjuvant chemotherapy or chemoradiotherapy has better clinical efficacy in patients with DC [7]. 5-FU, FOLFOX, and CAPOX were adopted as neoadjuvant regimens. However, there was scarce evidence of the utilization of S-1. S-1 is a relatively novel oral agent that has high efficacy and safety margin for advanced gastric and colorectal cancers [8, 9]. Today, S-1 is widely used for the treatment of gastrointestinal cancers and accepted as an alternative therapy to infused 5-FU, due to its administration convenience (oral intake) and less toxicity. Additionally, Fisherman et al. reported that chemotherapy including gemcitabine and irinotecan combinations appeared to have higher overall response rate compared to 5-FU-based regimen in a retrospective review of 113 patients with advanced small bowel adenocarcinoma [10]. We believe that GS was a suitable treatment regimen for the patient’s duodenal adenocarcinoma. However, the duration of this regimen is still unclear. In the present case, six courses of GS regimen were performed based on the preoperative treatment for pancreatic cancer in our institution.

Previous reports on the use of neoadjuvant chemotherapy with S-1 for patients with duodenal adenocarcinoma are listed in Table 1 [11–16]. A total of seven cases, including the present case, have been reported. The median age of the reported cases was 60 years (range, 48–72 years). Four patients were managed with S-1 and cisplatin, one patient was managed with S-1 with oxaliplatin in 1, one patient was managed with S-1 with gemcitabine, and one patient was managed with S-1 alone. Four patients had partial response treatment, and none of the patients experienced disease progression. The median follow-up period was 12 months (range, 6–23) with six patients alive. For patients with gastric cancer, neoadjuvant treatment with S-1 and cisplatin may also be performed [17]. According to the authors reported in Table 1, the regimen using S-1 and cisplatin was chosen because of its proximity to the stomach. Therefore, they followed neoadjuvant treatment for gastric cancer. However, there are no other reports that used S-1 with gemcitabine. In fact, duodenal adenocarcinoma is a relatively rare tumor. Thus, it is difficult to perform clinical trials on the usefulness of neoadjuvant chemotherapy. Further investigations such as multicenter study or using national database are necessary.

**Table 1:** Neoadjuvant chemotherapy with S-1 for duodenal adenocarcinoma

No.	Author	Year	Age	Sex	Regimen	Response	Prognosis	Period (month)
1	Egawa T	2008	60	M	S-1 + CDDP	PR	Alive	6
2	Kang SM	2009	48	M	S-1 + CDDP	PR	Dead	15
3	Mima K	2011	53	F	S-1 + CDDP	PR	Alive	12
4	Yamamoto S	2014	64	F	S-1	SD	Alive	18
5	Kanehira M	2017	71	M	S-1 + CDDP	SD	Alive	12
6	Zhang G-Y	2017	53	M	S-1 + oxaliplatin	CR	Alive	7
7	Present case	2022	72	F	S-1 + gemcitabine	PR	Alive	23

*CDDP* cisplatin, *PR* partial response, *SD* stable disease, *CR* complete response

Neoadjuvant chemotherapy with gemcitabine and S-1 was useful in reducing the size of the duodenal adenocarcinoma. This finding would aid physicians in managing patients who present with a similar presentation.

## Data Availability

All data generated or analyzed during this study are included in the published article.

## Abbreviations

DC: Duodenal carcinoma
CT: Computed tomography
GS: Gemcitabine and S-1
